# Pulmonary Hypertension Associated With Hyperthyroidism: A Case Report

**DOI:** 10.7759/cureus.30567

**Published:** 2022-10-21

**Authors:** Sakiko Kumata, Yoshitaka Hashimoto, Hiroshi Okada, Tatsuya Kawasaki

**Affiliations:** 1 Department of Cardiology, Matsushita Memorial Hospital, Moriguchi, JPN; 2 Department of Diabetes and Endocrinology, Matsushita Memorial Hospital, Moriguchi, JPN

**Keywords:** graves´disease, thyroid, treatment, pulmonary hypertension, hyperthyroidism

## Abstract

Pulmonary hypertension (PH) has various etiologies and its prognosis is unfavorable without appropriate treatment. We report a case in whom hyperthyroidism was considered as a cause of PH. A 32-year-old woman presented with exertional palpitation and dyspnea. Echocardiography showed an estimated systolic pressure in the pulmonary artery of 50 mmHg (reference value, 15 to 30) with no evidence suggestive of congenital heart disease or acquired heart disease. The level of D-dimer was normal and pulmonary perfusion scintigraphy was unremarkable. Thyroid function tests revealed a biochemically hyperthyroid state with elevated anti-thyroid peroxidase antibodies and thyroid stimulating hormone receptor antibodies, findings consistent with Graves’ disease. After the administration of treatment with potassium iodide and thiamazole, her symptoms and PH gradually abated and finally disappeared without any specific treatment for PH.

## Introduction

Pulmonary hypertension (PH) is characteristic of an elevation of pulmonary artery pressures for various etiologies, such as arterial abnormalities, left heart disease, or lung disease [1,2]. The prognosis is considered poor, especially in patients with pulmonary arterial hypertension, unless the condition is promptly diagnosed and appropriately treated. However, attention should be paid to several rare etiologies related to PH, e.g., hematologic, systemic, or metabolic disorders [1,2]. We report a case of PH in whom hyperthyroidism was considered as a cause of PH and, after the initiation of thyroid treatment, PH resolved without any specific treatment for PH.

## Case presentation

A 32-year-old woman presented with a one-to-two-year history of palpitation and dyspnea on effort. The symptoms had gradually deteriorated, but she thought that they were the result of mental problems. She was a nurse working in a major hospital, and her medical history was unremarkable. At an annual checkup, she was found to have abnormal electrocardiography, which made her decide to seek medical care. She stated that she had difficulty climbing the stairs to the second floor. The patient did not take any medication, including over-the-counter supplements, at the time of presentation. She did not drink or smoke and had no known allergies.

On examination, she was not lethargic. Her blood pressure was 143/63 mmHg, her pulse rate was 96 beats per minute, her body temperature was 36.3 °C, and her oxygen saturation level was 98% while she was breathing ambient air. Both lungs were clear on auscultation and a cardiac examination produced normal findings. There was no edema in her legs, and except for a diffuse goiter, the remainder of the examination was normal.

Electrocardiography showed frequent premature atrial contractions with an enlargement of the right atrium (i.e., high voltage in the inferior leads, 0.3 mV), although electrocardiographic findings obtained one year earlier were normal. A chest radiograph exhibited normal findings. Her complete blood cell count was normal, as were her electrolyte balance, C-reactive protein level, and renal and liver function tests. The level of brain natriuretic peptide was 25.3 pg/mL (reference value, ≤ 18.4). Echocardiographic examination showed normal chamber sizes and ventricular function. Systolic pulmonary blood pressure was estimated at 50 mmHg. There was no evidence suggesting congenital heart disease or acquired heart disease. A diagnosis of PH from unknown causes was made.

The level of D-dimer was normal and no abnormalities were found on pulmonary perfusion scintigraphy or duplex ultrasound investigation of the veins of the lower extremities. The level of antinuclear antibodies was ×40 (reference value, < 40) and the level of anti-deoxyribonucleic acid antibodies was 2.0 IU/mL (reference value, ≤ 6). Thyroid function tests revealed a biochemically hyperthyroid state (Table 1). The level of positive anti-thyroid peroxidase antibodies was 316 IU/mL (reference, < 3.3) and the level of thyroid-stimulating hormone receptor antibodies was 161 IU/L (reference, < 1.0), findings consistent with Graves’ disease. Treatment with potassium iodide at a dose of 0.05 g daily and thiamazole at a dose of 15 mg daily was initiated. Her symptoms abated in response to decreases in pulmonary artery systolic pressure as well as thyroid hyperactivity (Table 1). She was doing well without any symptoms for more than three months after the disappearance of PH.

**Table 1:** Clinical course of the patient

Variable	Reference range	At presentation	One month later	Two months later	Three months later	Four months later	Five months later	Seven months later	Eight months later
Thyroid function tests									
Free T3 (pg/mL)	2.6-5.1	32.5	6.75	5.47	4.38	5.90	2.79	2.40	2.93
Free T4 (ng/dL)	1.00-1.81	7.77	1.99	1.75	1.35	1.99	0.77	0.54	0.75
Thyroid stimulation hormone (μU/mL)	0.27-4.20	0.01	0.01	0.01	0.01	0.01	0.01	0.84	0.43
Medication									
Potassium iodide (g/day)	0.05	0.05	0.05	0.05	0	0.05	0.05	0.05	0.05
Thiamazole (mg/day)	15	0	15	15	15	15	10	5	5
Echocardiography									
Peak tricuspid regurgitation velocity (m/s)	-	3.4	2.7	2.6	-	2.2	-	-	2.3
Tricuspid regurgitation pressure gradient (mmHg)	-	46	29	27	-	19	-	-	21
Pulmonary artery systolic pressure (mmHg)	15-30	50	35	35	-	25	-	-	25

## Discussion

The present patient presented with worsening palpitation and dyspnea and was found to have PH. Her laboratory assessment and imaging examination were unremarkable except for a hyperthyroid state due to Graves’ disease. After the initiation of thyroid treatment, her symptoms and PH gradually improved and finally disappeared without any specific treatment for PH.

PH is classified into five groups based on etiologies and pathophysiologic mechanisms: Group 1-pulmonary arterial hypertension; Group 2-PH due to left heart disease; Group 3-PH due to lung disease or hypoxia; Group 4-chronic thromboembolic PH); and Group 5-PH due to unclear multifactorial mechanisms [1]. In the present case, the possibilities of classification into Groups 2, 3, and 4 were ruled out, although no invasive examination was performed to measure pulmonary artery pressure, pulmonary capillary wedge pressure, or pulmonary vascular resistance. Given her clinical course, PH in the present case was considered as Group 5 associated with metabolic disorders (thyroid disorders), as it is rare for pulmonary arterial hypertension (Group 1) to mitigate without any specific treatment for PH [2].

It is reported that PH may be related to thyroid disease during its course. In a retrospective study of 356 consecutive PH patients and 698 sex-matched control subjects without PH, the prevalence of thyroid disease was significantly higher in patients with PH (24%) than in the control (15%) [3]. Evaluation of thyroid function in PH patients may be warranted to detect and assess coexisting thyroid disease, although it may sometimes be missed. In a systematic review that included 589 patients with PH and hyperthyroidism, the etiologies were Graves’ disease (66.7%) as shown in our case, toxic multinodular goiter (16.8%), and thyroiditis (0.8%) [4]. It is important to note that not only hyperthyroidism but also hypothyroidism can be related to PH. The prevalence of PH was 35% to 47% among patients with hyperthyroidism, whereas 10% to 24% of patients with primary PH had hypothyroidism [5]. Thus, a periodic thyroid test (e.g., once every one to two years) is recommended in patients with PH [2].

It remains unclear whether the association between PH and thyroid diseases is incidental or causative. Although patients’ characteristics between the two conditions, such as younger female predominance, may substantially overlap, the influence of thyroid hormones on the cardiovascular system should also be noted. Several studies reported the effects of both hyperthyroidism and hypothyroidism on the occurrence of increased vascular pulmonary arterial pressure [5]. The precise mechanism is not fully investigated and may be multifactorial. The proposed mechanisms are the direct effects of thyroid hormones on pulmonary vascular proliferation, the chronotropic effects of hormones on the cardiovascular system, and autoimmune-mediated endothelial dysfunction [4,5]. An echocardiographic examination showed that female patients with hyperthyroidism, like the present case, had elevated cardiac output and pulmonary vascular resistance [6]. In patients with hyperthyroidism, estimated systolic pulmonary artery pressure was positively correlated to the levels of free thyroxine and the duration of hyperthyroidism [7]. In the present case, the estimated pulmonary systolic pressure decreased as a function of a decrease in the levels of free thyroxine.

Data from randomized trials are lacking regarding the effects of the treatment of thyroid diseases on PH. In a prospective study consisting of 17 patients with PH and hyperthyroidism, all patients except one normalized their pulmonary artery systolic pressures with thyroid treatment consisting of methimazole (n = 13), total thyroidectomy (n = 2), and 131-I treatment (n = 1) at least nine months after achieving a stable euthyroid status [8]. Similarly, in the present patient, the pulmonary artery systolic pressures as assessed by echocardiography started decreasing after the initiation of thyroid treatment and finally normalized two months before euthyroid status. Although treatment delay would not be preferred in patients with PH, a thyroid-first strategy may be useful in some cases. Further research is needed to examine the implications for patients with PH and hyperthyroidism.

## Conclusions

We experienced a case of PH associated with metabolic disorder or Graves’ disease and successfully treated PH with thyroid treatment. Our case highlights the importance of acknowledging that various etiologies need to be considered in patients with PH. It is worth noting that thyroid disease can be a cause of PH and that PH may be managed with thyroid treatment.
